# Outcome and Prognostic Indicators of Patients with Hematopoietic Stem Cell Transplants Admitted to the Intensive Care Unit

**DOI:** 10.1155/2009/917294

**Published:** 2009-09-15

**Authors:** Thanh N. Huynh, S. Sam Weigt, John A. Belperio, Mary Territo, Michael P. Keane

**Affiliations:** ^1^Division of Pulmonary and Critical Care Medicine, Department of Medicine, David Geffen School of Medicine at UCLA, Los Angeles, CA 90095-1690, USA; ^2^Division of Hematology and Oncology, Department of Medicine, David Geffen School of Medicine at UCLA, Los Angeles, CA 90095-1690, USA; ^3^Department of Medicine, St Vincent's University Hospital and University College Dublin, Dublin 4, Ireland

## Abstract

The prognosis of patients with hematopoietic stem cell transplants (HSCTs) who require admission to the intensive care unit (ICU) has been regarded as extremely poor. We sought to re-evaluate recent outcomes and predictive factors in a retrospective cohort study. Among the 605 adult patients that received an HSCT between 2001 and 2006, 154 required admission to the ICU. Of these, 47% were discharged from the ICU, 36% were discharged from the hospital, and 19% survived 6 months. Allogeneic transplant, mechanical ventilation, vasopressor-use, and neutropenia were each associated with increased mortality, and the mortality of patients with all four characteristics was 100%. Hemodialysis was also associated with increased mortality in a Kaplan-Meier analysis but did not appear important in a multivariate tree analysis. A final Cox model confirmed that allogeneic transplant, mechanical ventilation, and vasopressor-use were each independent risk factors for mortality in the 6 months following ICU admission.

## 1. Introduction

The use of hematopoietic stem cell transplant (HSCT) has become the standard of care for many types of hematologic malignancies, including acute and chronic leukemias and lymphomas. Unfortunately, HSCT is often associated with severe complications, including infection, respiratory failure from multiple etiologies, graft versus host disease, bleeding, sepsis, and multiorgan failure [1–4]. These complications can occur acutely, especially during the period of neutropenia when patients are profoundly immunocompromised or at a later time after hospital discharge [5]. Such complications often require admission to the intensive care unit (ICU) for higher level of care. The reported range of admission to the ICU is between 11% and 40% of all HSCT recipients [5, 6]. This range is likely due to the differences in acuity level required for ICU admission and the differences in percent of allogeneic transplants at different institutions.

Previous studies have shown that HSCT patients who require ICU admission often face a very poor prognosis, with mortality ranging from 54% to 92% and always above 80% when mechanical ventilation is required [6–9]. While the decision to limit aggressive care in HSCT patients is often difficult, identification of prognostic factors can aid in the counseling of patients and families so that the use of futile measures can be minimized.

Over the past few decades, advances in the procedure and care of patients after transplantation may have improved survival. Given the significant financial and emotional costs associated with ICU care, it is important to be able to provide clinicians, patients, and families with the most current estimates of mortality and prognostic factors for HSCT patients who require ICU admission. In this study, we evaluated the clinical characteristics of HSCT patients who required ICU admission, their survival rates, and the characteristics that predict adverse outcome. Furthermore, we compared the survival of patients who were admitted to the ICU *during* their initial hospitalization for HSCT to the survival of patients who required ICU care when *readmitted* after they have been discharged status posttransplant.

## 2. Patients and Methods

### 2.1. Patients and Data

With IRB approval, we retrospectively reviewed all adult patients who had received HSCTs who were admitted to the University of California at Los Angeles Medical Center intensive care unit during the five-year period between July 2001 and June 2006. Clinical charts were reviewed to document each patient's age, gender, type of malignancy, type of HSCT (allogeneic versus autologous), underlying disease, reason for ICU admission, and length of ICU stay. We documented whether the patient was neutropenic, which is defined by an absolute neutrophil count (ANC) of <500 during any point during their ICU admission and whether the patient had signs of GVHD. Organ failure was assessed by establishing whether the patient required invasive mechanical ventilation, vasopressors, or hemodialysis during their ICU stay. In our institution, the use of organ failure support, such as mechanical ventilation after intubation, vasopressor support, or continuous renal replacement therapy, is restricted to the ICU.

Survival to discharge from the ICU, from the hospital, and up to 6 months after ICU admission was assessed. For patients who had more than one admission to the ICU, only the first admission was used for analysis. Patients who were discharged from the ICU and transferred back to the ward, regardless of ultimate outcome, were considered to have survived the ICU.

### 2.2. Statistical Analysis

Data were reported as proportions, means (±SD), or medians (intraquartile range) where they are appropriate and as indicated within the text and tables. ICU, Hospital, and 6-month survival proportions in autologous and allogeneic HSCT patients were compared with chi-square tests. The predetermined primary objective of this study was the identification of independent prognostic variables for survival in the 6 months following ICU admission in HSCT patients. Potential prognostic variables were first screened by Kaplan-Meier analysis. Promising variables (*P*-value <.10 by Mantel-Cox log-rank test) were then included in a tree model to inform about associations and a variable's level of importance. The tree was constructed using the partition modeling feature included in the JMP 7 software package. The software was allowed to determine the best next cut, and additional cuts were not made to a branch with a sample size less than 30. Finally, guided by the rule of 10, the “best” 3 variables were determined from the first 3 cuts in the tree model and included in a final multivariate Cox Proportional Hazards model of survival for the 6 months following ICU admission.

In additional exploratory studies, the study cohort was further divided and analyzed in three subgroups: the HSCT Admission group consisted of patients who were admitted to the ICU during the course of their *initial* hospitalization for hematopoietic transplant (*n* = 70), the Early Readmission group was patients who were discharged from the hospital status post-HSCT but who require ICU admission within 100 days of their transplant (*n* = 33), and the Late Readmission group was HSCT patients who required ICU admission more than 100 days after their transplant (*n* = 51). Six-month survival rates were compared among the three groups.

## 3. Results

### 3.1. HSCT and ICU Admission

UCLA performed 605 adult HSCT transplants (400 autologous, 205 allogeneic) during this study period. Three patients admitted to the ICU for monitoring after elective surgeries were excluded from our study. After the exclusion of these patients, 154 patients, or 25% of the total adult HSCT cohort, required 179 ICU admissions. Admission to the ICU was more common after allogeneic transplant (46%) than autologous transplant (15%). During the initial hospitalization for HSCT, 70 patients (11.6% of the total cohort) required admission to the ICU. Similarly, during the initial hospitalization for HSCT, ICU admission was more common after allogeneic transplant (22.4%) than autologous transplant (6%).

### 3.2. HSCT Patient Characteristics

**Figure 1:**
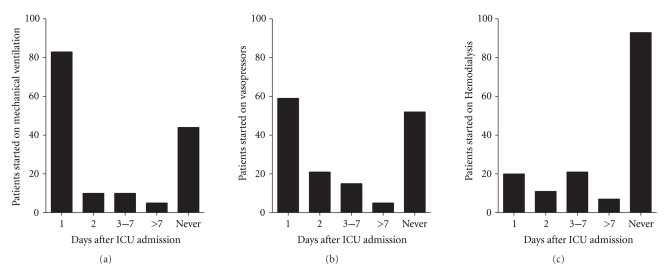
(a) Number and timing of patients receiving mechanical ventilation following ICU admission. (b) Number and timing of patients receiving vasopressor following ICU admission. (c) Number and timing of patients receiving hemodialysis after ICU admission.

The characteristics of these 154 HSCT patients admitted to the ICU are summarized in Table 1. There were 91 males and 63 females. Overall, the top three underlying diseases that necessitated HSCT were acute myeloid leukemia (29%), non-Hodgkin's lymphoma (21%), and acute lymphoblastic leukemia (16%). The average age was 44 (range was 18–77), and the median length of ICU stay was 5 days (IQR 2, 11). Sixty-five patients (68%) had signs of GVHD, and 66 patients (43%) were neutropenic at one point during their ICU admission. Mechanical ventilation was required in 110 patients (71%), vasopressor-use was required in 104 patients (66%), and dialysis was required in 63 patients (41%). The majority of patients who needed mechanical ventilation or vasopressors during their ICU admission required initiation of these interventions on their first ICU day, while initiation of HD generally occurred during the first week or not at all (Figure 1). During this study, the initial vasopressor choice was norepinephrine. Neosynephrine or vasopressin may have been added as second agents when needed. The reasons for ICU admissions are summarized in Table 2. The most frequent reasons for ICU admission were respiratory failure and hemodynamic instability. Neurologic conditions that necessitated ICU admission included seizures, profound altered mental status, or subdural monitoring.

### 3.3. Outcome of HSCT Patients Admitted to the ICU

**Figure 2:**
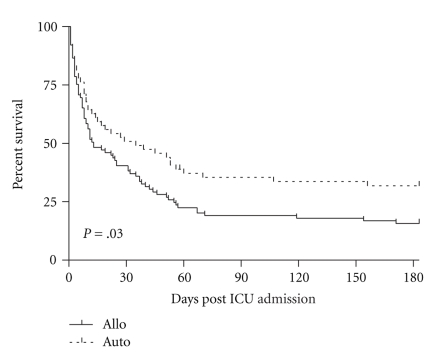
Survival in the 6 months following ICU admission is generally better in autologous HSCT patients compared to allogeneic HSCT patients.

Among the 154 HSCT patients who required ICU admission, 47% (72 patients) were discharged from the ICU, 36% (55 patients) were discharged from the hospital, and 19% (30 patients) were alive at 6-month followup. Five patients discharged from the ICU were discharged for hospice arrangement and terminal care. In the 6 months following ICU admission, survival was generally better in autologous HSCT patients than in allogeneic HSCT patients (Figure 2). A greater proportion of autologous than allogeneic HSCT patients survived to ICU discharge (61% versus 38%, *P* = .005), hospital discharge (56% versus 22%, *P* < .001) and for at least 6 months after the ICU admission (31% versus 13%, *P* = .007).

### 3.4. Prognostic Characteristics

**Figure 4:**
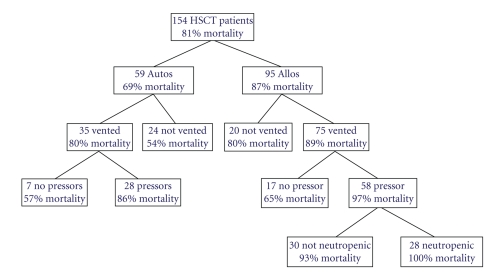
6-month mortality model for 154 HSCT patients who were admitted to the ICU. Out of the 154 HSCT patients who were admitted to the ICU, 81% were not alive 6 months after ICU admission. Patients who had all 4 prognostic indicators (allogeneic transplant, mechanical ventilation, vasopressor-use, and neutropenia) had 100% mortality.

We examined the impact of potential prognostic factors by comparison of Kaplan-Meier survival curves for the 6 months following ICU admission (Figure 2). A requirement of mechnical ventilation, vasopressor-use, hemodialysis, or the presence of neutropenia was each associated with increased mortality when examined alone. Because these variables often occurred together in the same patient, we explored for interactions and the level of importance in a tree model (Figure 4). In this model, each negative prognostic factor increased mortality, and patients with the 4 most important prognostic factors (allogeneic transplant, mechanical ventilation, vasopressor-use, and neutropenia) had 100% mortality. Hemodialysis did not factor in this model.

Based on the tree model, allogeneic transplant, mechanical ventilation, and vasopressor-use were determined to be the “best” prognostic variables for predicting the risk of mortality in the 6 months after ICU admission, and these variables were included in a multivariate Cox proportional hazards model. In this model, allogeneic HSCT, mechanical ventilation, and vasopressor requirement were each independent risk factors for mortality (Table 3).

### 3.5. Timing of ICU Admission and Outcome

**Figure 5:**
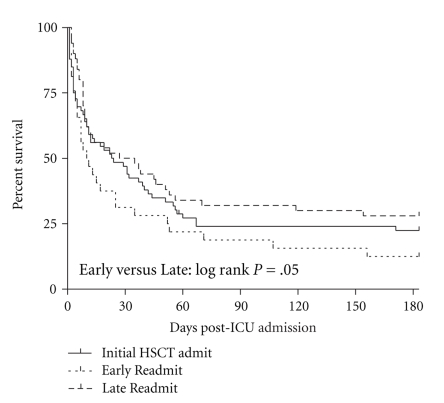
Kaplan-Meier 6-month survival curve separated by timing of ICU admission. HSCT Admission Group is patients who were admitted to the ICU during the course of their initial admission for HSCT, Early Readmission Group is patients who have been discharged from the hospital since their HSCT but still within 100 days of their transplant, and Late Readmission Group is patients who have been discharged from the hospital since their HSCT but who are greater than 100 days out from their transplant.

When the study population was separated by the timing of ICU admission, we found that 70 patients were admitted to the ICU during the course of their *initial* hospitalization for HSCT (HSCT Admission Group), 33 patients were admitted to the ICU after being discharged but who were still within 100 days of their transplant (Early Readmission Group), and 51 patients required ICU admission after more than 100 days after their transplant (Late Readmission Group). In the HSCT Admission Group, 49% (34) were discharged from the ICU, 34% (24) were discharged from the hospital, and 20% (14) were alive at 6 months after ICU admission. The Early Readmission Group had the lowest ICU, hospital, and 6-month survival (36%, 21%, and 12%, resp.). The Late Readmission Group's survival for the ICU, hospital, and at 6 months was 51%, 47%, and 24%, respectively. In general, survival in the 6 months following ICU admission was significantly worse in the Early Readmission Group than in the Late Readmission Group (Figure 5).

**Figure 6:**
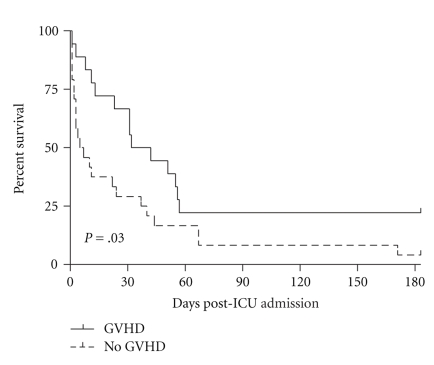
Kaplan meier curves during 6 months after admission to the ICU depending on the presence of GVHD for HSCT Admission Group (log rank test, *P* = .03).

Among allogeneic HSCT patients in the HSCT Admission Group, the presence of GVHD was associated with improved 6-month survival (Figure 6). Most patients in this group (75%) were admitted to the ICU within two weeks of their transplant, and thus were unlikely to have engrafted in order to develop GVHD. The presence of GVHD during later ICU admissions and for the total cohort had no significant effect on survival (data not shown).

## 4. Discussion

Although HSCT is the treatment of choice for many types of hematologic malignancies, it continues to be associated with considerable morbidity and mortality. Early studies have reported survival rates less than 20% and often approaching 0% once mechanical ventilation become necessary [6, 7]. An earlier report at our own institution demonstrated that only 1 out of 40 bone marrow transplant patients who were intubated for acute respiratory failure survived to ICU discharge [10]. That one individual developed recurrent leukemia and died within 10 months. With such poor outcomes, the use of costly aggressive interventions for these patients has been questioned [7, 10, 11]. However, more recent studies have suggested that the prognosis of HSCT patients requiring ICU admission has improved over the last two decades [5, 12, 13], and our data support this conclusion. In this study, the overall ICU, hospital, and 6 month survival was 47%, 36%, and 19%, respectively. For mechanically ventilated patients, the survival was 30%, 22%, and 13%, respectively. The reason for this improvement is likely multifactorial. The wider use of peripheral cells as the source of stem cells has been associated with shorter time to engraftment [14], and faster platelet and neutrophil recovery likely decrease the risks of opportunistic infections and life-threatening bleeding. Price et al. were able to demonstrate that recipients of peripheral blood stem cell transplants have a more favorable outcome than bone marrow transplant patients should they require mechanical ventilation [8]. In addition, the use of less myeloablative conditioning has been shown to reduce posttransplant morbidity [15]. It is also possible that there has been improvement in critical care management with the advent of early goal directed therapy and improved mechanical ventilation strategies for acute lung injury [16, 17].

Other recent studies have reported survival after ICU admission in HSCT patients that is better than in our study. Soubani et al. report a 61% ICU-survival, and Afessa et al. report a 54% hospital survival [5, 6]. The different findings between these and our own study may be a result of variable utilization of ICU-level care at different institutions and differences in baseline patient population. Ninety-three percent of our study population had evidence of organ failure requiring dialysis, mechanical ventilation, or hemodynamic support, and it is possible that our results represent the outcome of more critically ill patients as well as high-risk patients receiving HSCTs. In addition, 62% of this study's population had an allogeneic transplant, which is known to put patients at higher risk as it is frequently complicated by GVHD and prolonged periods of immunosuppression [18]. This is further supported by our data that show a 4-fold greater incidence of ICU admission in allogeneic HSCT patients as compared to autologous HSCT patients.

The principal aim of our study was to identify the characteristics of HSCT patients admitted to the ICU that predict poor outcome. Multiple other studies have sought to identify the variables that might predict worse outcome [5, 7, 9, 11, 14, 18–20]. Unfortunately, there has not been consensus, and Jackson et al. and Evison et al. found that pre-ICU admission variables could not be used to predict mortality [21, 22]. In our study, we found that allogeneic transplant, mechanical ventilation, neutropenia, vasopressor-use, and hemodialysis were each significantly associated with decreased 6-month survival when examined individually. When these variables were included together in a tree model of 6-month survival, allogeneic transplant, mechanical ventilation, vasopressor-use, and neutropenia were each associated with increased mortality. In this model, the source of transplant was the first cut made and therefore appears to be the most important determinant of 6-month mortality, with 69% mortality in autologous HSCT patients and 87% mortality in allogeneic HSCT patients admitted to the ICU. This result is not surprising in view of the fact that allogeneic transplants are associated with longer engraftment periods, longer periods of neutropenia, and are often complicated by GVHD, which then require profound and prolonged periods of immunosuppression. While hemodialysis was associated with increased mortality in the univariate analysis, it was surprisingly not a factor in the multivariate tree model. Hemodialysis was never an indication for ICU admission alone and therefore occurred in the presence of respiratory failure or hemodynamic instability. This finding suggests that the requirement for hemodialysis does not add to the risk of other associated negative prognostic variables. However, this conclusion is admittedly limited by the small number of patients who required hemodialysis in this study. Importantly, a multivariate Cox proportional hazards model confirmed that allogeneic transplant, ventilator requirement, and vasopressor-use were each independent risk factors for mortality in the 6 months following ICU admission.

We also explored the effect of timing of ICU admission in subgroup analyses. We find that patients in the Early Readmission group, which are patients who have been discharged status post-HSCT but who are within 100 days of their transplant, have a worse 6-month outcome than those admitted later. Only 12% of the Early Readmission Group survives 6 months after ICU admission. To our knowledge, this is the first study to evaluate patients who have been discharged from the hospital after HSCT, divide them into groups in this manner, and find that patients who require ICU admission soon after discharge have a worse 6-month prognosis. Soubani et al. found that the majority of HSCT patients who require ICU level care do so within the first 100 days of their transplant [5], and we find that these patients also have a worse outcome, particularly if they were already discharged from the hospital after their transplant. We suspect that the patients in the Early Readmission Group may be especially vulnerable because they remain at high risk of complications but do not have access to immediate care when they start to deteriorate. Our result emphasizes the need to be attuned to the special risks and complications of patients who have had a recent HSCT and to be aggressive in their treatment especially when these patients require readmission, as they can deteriorate rapidly.

Previous studies have shown that GVHD, either through direct complications or by requiring immunosuppression that increase infection susceptibility, is an important risk factor in predicting mortality in allogeneic transplants [23]. Afessa et al. found that GVHD was associated with increased mortality in patients admitted to the ICU [13]. In our study, GVHD was surprisingly associated with improved survival in the HSCT Admission Group. We suspect that in this group, the presence of GVHD was a marker for engraftment and for a later time point posttransplantation, both of which may be associated with a better prognosis for survival after an ICU admission. This hypothesis is supported by the finding that, in the HSCT Admission group, patients with GVHD were admitted to the ICU at a significantly later time-point posttransplantation than those without GVHD (14 days, intraquartile range 11–31 days versus 9 days, intraquartile range 2–14 days, resp.; *P* = .003). In the other subgroups, and for the entire cohort, GVHD had no significant effect on survival.

Our study has shown that the outcome of HSCT patients who require ICU-level care is not as poor as previously described. Our own institution has seen an improvement in the survival of HSCT patients who require mechanical ventilation. However, although ICU-level care may be appropriate, mortality does remain high in this patient population, particularly among those who had an allogeneic transplant, require vasopressors or mechanical ventilation, or are neutropenic. As seen by our tree model (Figure 4), each of these factors negatively affects the prognosis for 6-month survival in HSCT patients admitted to the ICU. Furthermore, the reported ICU survival rate of the unselected, heterogeneous ICU population is about 70% [24] and thus better than that of HSCT patients (47%). Therefore, clinicians need to be encouraged to use studies like ours to provide patients with accurate estimates of outcome and initiate early discussions regarding advance directives. As recommended by Crawford and Rubenfeld in the original study regarding hematopoietic transplant and ICU care, “the goal of HSCT is to cure the underlying condition and return the patient to an acceptable quality of life. When these goals are no longer attainable, intensive life support should cease” [7]. Nonetheless, with advances in technology and medicine, both the effectiveness of ICU therapeutic modalities and hematopoietic transplantation may improve. It is important that we continue to monitor the effects of these changes on outcome over time.

## Figures and Tables

**Figure 3:**
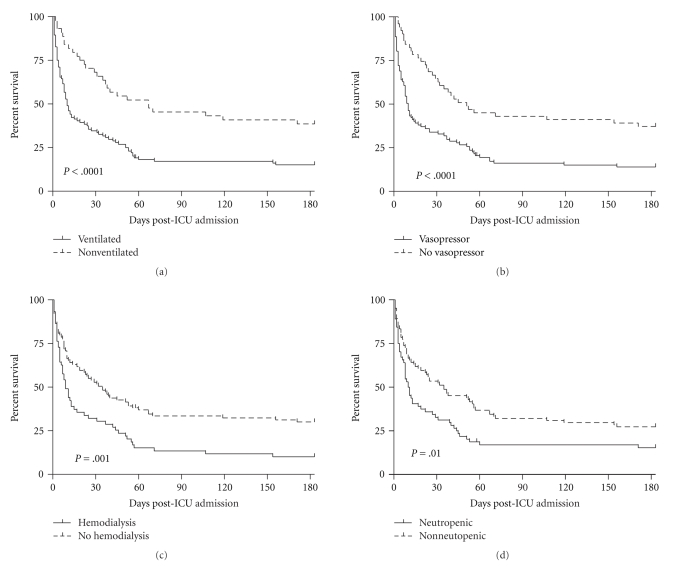
Kaplan Meier survival curves for 6 months after admission to ICU depending on requirement for ventilation, vasopressor-use, hemodialysis, and the presence of neutropenia.

**Table 1:** Clinical characteristics of HSCT patients requiring ICU admission.

Characteristics	HSCT patients requiring ICU (*n* = 154)
ICU admissions, *n*	179
Type of transplant, *n* (%)	
Autologous	59 (38%)
Allogeneic	95 (62%)
Age, mean (SD)	44 (±14)
Days post-HSCT, median (IQR)	48 (11, 208)
ICU days, median (IQR)	5 (2, 11)
Gender, *n * (%)	
Male	91 (59%)
Female	63 (41%)
Underlying disease, *n* (%)	
Acute myeloid leukemia	45 (29%)
Non-hodgkin's lymphoma	32 (21%)
Acute lymphoblastic leukemia	25 (16%)
Hodgkin's lymphoma	17 (11%)
Multiple myeloma	9 (6%)
Chronic myeloid leukemia	7 (4.5%)
Breast cancer	4 (2.6%)
Myelodysplastic syndrome	4 (2.6%)
Aplastic anemia	3 (1.9%)
Paroxysmal nocturnal hemaglobinuria	2 (1.3%)
Chronic lymphoblastic leukemia	2 (1.3%)
Other	4 (2.6%)
Source of stem cell	
Peripheral stem cell transplant	139 (90%)
Bone marrow transplant	14 (9.0%)
Cord blood	1 (0.6%)
GVHD, *n* (% of Allo HSCT)	65 (68%)
Neutropenia	66 (43%)
Mechanical ventilation	110 (71%)
Vasopressors	104 (66%)
Dialysis	63 (41%)

**Table 2:** Reason for ICU admission.

Reason for ICU admission	Number of patients (%)
Respiratory failure	79 (51%)
Hemodynamic instability	35 (24%)
Both respiratory failure and hypotension	21 (14%)
Neurologic condition	13 (8.4%)
Gastrointestinal bleeding	5 (3.3%)
Observation for severe, refractory thrombocytopenia	1 (0.65%)

**Table 3:** Predictors of 6-month Mortality.

	Multivariate model		
	Risk Ratio	95% CI	*P*-value
Allo transplant	1.51	1.04–2.25	.031
Ventilated	1.87	1.21–2.96	.004
Vasopressors	2.07	1.38–3.19	.003
